# Emoji-SP, the Spanish emoji database: Visual complexity, familiarity, frequency of use, clarity, and emotional valence and arousal norms for 1031 emojis

**DOI:** 10.3758/s13428-022-01893-6

**Published:** 2022-06-17

**Authors:** Pilar Ferré, Juan Haro, Miguel Ángel Pérez-Sánchez, Irene Moreno, José Antonio Hinojosa

**Affiliations:** 1 Departament de Psicologia and CRAMC, Universitat Rovira i Virgili, Carretera de Valls, s.n, 43007 Tarragona, Spain; 2 Departamento de Psicología Básica y Metodología, Universidad de Murcia, Murcia, Spain; 3 Departamento de Psicología Experimental, Procesos Cognitivos y Logopedia, Universidad Complutense de Madrid, Madrid, Spain; 4 Instituto Pluridisciplinar, Universidad Complutense de Madrid, Madrid, Spain; 5 Centro de Investigación Nebrija en Cognición, Universidad Antonio de Nebrija, Madrid, Spain

**Keywords:** Emoji, Normative study, Subjective ratings, Visual complexity, Familiarity, Frequency of use, Clarity, Emotional valence, Emotional arousal

## Abstract

This article presents subjective norms for 1031 emojis in six dimensions: visual complexity, familiarity, frequency of use, clarity, emotional valence, and emotional arousal. This is the largest normative study conducted so far that relies on subjective ratings. Unlike the few existing normative studies, which mainly comprise face emojis, here we present a wide range of emoji categories. We also examine the correlations between the dimensions assessed. Our results show that, in terms of their affective properties, emojis are analogous to other stimuli, such as words, showing the expected U-shaped relationship between valence and arousal. The relationship between affective properties and other dimensions (e.g., between valence and familiarity) is also similar to the relationship observed in words, in the sense that positively valenced emojis are more familiar than negative ones. These findings suggest that emojis are suitable stimuli for studying affective processing. Emoji-SP will be highly valuable for researchers of various fields interested in emojis, including computer science, communication, linguistics, and psychology. The full set of norms is available at: https://osf.io/dtfjv/.

## Introduction

The way people communicate with each other has undergone a major transformation in the last 20 years due to technological progress. Widespread use of the Internet and mobile devices has led to the emergence of new forms of written communication. Despite its many advantages, however, one limitation of computer-mediated communication (CMC) is the absence of nonverbal cues such as gestures, prosody, and facial expressions. This can result in poorer transmission of information (Archer & Akert, 1977; Walther, 1996). One strategy to overcome this limitation and improve the efficiency of communication has been the development of new nonverbal cues such as emoticons, emojis, and stickers. Emoticons are symbols created with punctuation marks, letters, and numbers to represent mainly facial expressions, emotions, and abstract concepts. Emojis are colored graphic symbols with predefined names and code (Unicode) covering a wide range of areas. Apart from those representing facial expressions, emotions, and abstract concepts (like emoticons), emojis also comprise symbols for animals, plants, body parts, clothes, drinks, family, food, professions, sports, and vehicles, among others (Rodrigues et al., 2018). Many referents can also be represented by stickers (oversized and complex illustrations or animations used as thematic sets to communicate emotions, social situations, opinions, and intentions, De Seta, 2018; Konrad et al., 2020; Lee et al., 2016), which have appeared on the scene more recently and are mainly used in communicative contexts that involve close social relationships (Konrad et al., 2020).

Of the nonverbal cues used in CMC, emojis are the most widely used and the most extensively studied from the scientific point of view (Bai et al., 2019). First introduced in Japan 30 years ago, their number has increased continuously. In fact, over 3000 emojis are now available (Unicode version 14.0: https://unicode.org/emoji/charts/full-emoji-list.html). Emojis have two main communicative functions (Kaye et al., 2016). First, they contribute to the emotional content of messages. Indeed, several studies have shown that many emojis carry an affective meaning (e.g., Jaeger et al., 2019; Novak et al., 2015; Riordan, 2017; Shoeb et al., 2019), while others have shown that messages that include emojis are perceived to be emotionally more intense than those that do not (Erle et al., 2021). Second, emojis reduce written-message ambiguity. For instance, when Novak et al. (2015) asked participants to rate the valence of tweets with or without emojis, they found greater agreement across raters for the former than for the latter. Riordan (2017), on the other hand, showed that participants rated ambiguous messages (i.e., they contained words with multiple meanings, or homonyms, such as “shot”) as less ambiguous when they included emojis. Emojis have also been shown to facilitate the recognition of conveyed indirect meanings of sentences (Holtgraves & Robinson, 2020). Finally, in addition to contributing to the emotional content of messages and helping to clarifying meaning, emojis can simply be used for fun or social purposes (see Tang & Hew, 2019, for a review).

The pervasiveness of emojis in CMC has generated much scientific interest in these nonverbal communicative cues. Indeed, in recent years much research has been conducted in such diverse fields as computer science, communication, marketing, medicine, education, linguistics, and psychology, among others (see Alattar, 2021; Aldunate & González-Ibáñez, 2017; Bai et al., 2019, Evans, 2017, and Tang & Hew, 2019, for reviews).

One line of research focuses on naturalistic data to establish the variables that influence emoji use (e.g., Novak et al., 2015). It has been shown that emoji use is more frequent in pleasant (Derks et al., 2007) and informal (Rosen et al., 2010) contexts than in unpleasant and formal ones. Emoji use is also affected by individual differences such as age, gender, psychological traits, and mood. Indeed, emoji use decreases as the age of users increases (e.g., Prada et al., 2018; Settanni & Marengo, 2015), which might be an indicator of generational differences. Moreover, females use emojis more frequently than males and have a more positive attitude towards them (Jones et al., 2020; Prada et al., 2018), while males use a wider range of emojis than females (Tossell et al., 2012). In addition, females perceive emojis as clearer, more meaningful, and more familiar than men (Rodrigues et al., 2018). The perception of recipients is also affected by the gender of the sender: when women send messages with affectionate emojis, they are perceived as more attractive than men, whereas when men send messages with friendly emojis, they are perceived as more attractive than women (Butterworth et al., 2019). With regard to psychological traits, extraversion has been correlated with emoji use (Hall & Pennington, 2013), especially in relation to positive emojis (Li et al., 2018). Mood also has an effect, since people use emojis more often when they are in a good mood (Konrad et al., 2020). Emojis can also influence the receiver’s mood, as has been demonstrated by Das et al. (2019), who showed that consumers experience a more positive affect when presented with advertisements that include emojis. Finally, cultural differences have also been identified in emoji use, with users from certain cultures using more emojis representing positive/negative emotions than those from other cultures (Cheng, 2017, Xuan et al., 2016; see also Guntuku et al., 2019; Lin & Chen, 2018, for other cultural differences).

From a different line of inquiry, a more experimentally oriented approach has examined emoji affective processing (e.g., Comesaña et al., 2013; Fischer & Herbert., 2021; Kaye et al., 2021, 2022; Kerkhof et al., 2009). In one of the first studies conducted in this field, Comesaña et al. (2013) used an affective priming paradigm and found that masked emojis, but not words, facilitated the processing of affectively congruent words presented immediately afterwards. The authors interpreted these results as indicating a privileged affective processing of emojis with respect to words. The capacity of emojis to prime affectively congruent words at a neural level has also been evidenced recently (Yang et al., 2021). Other studies have demonstrated that embedding emojis in a text impacts its affective processing (Pfeifer et al., 2022). A related line of research has compared the processing of emojis to other kinds of stimuli, such as words or faces. The results have shown a similarity of the response to emojis and facial expressions (e.g., Gantiva et al., 2020; Weiβ et al., 2019). It should be noted, however, that other studies have failed to find an exact correspondence between face and emoji processing. For instance, Kaye et al. (2021) found that participants responded faster to emojis than to faces (although emotion did not further modulate the processing of emojis or faces in this study).

The study of semantic processing of emojis embedded in text is also of great interest. Indeed, considering that emojis may function in written discourse in similar ways as nonverbal cues in face-to-face communication, and that they also contain their own linguistic and semantic properties, it is logical to expect an influence of emojis in the processing of the accompanying text (Robus et al., 2020). There are only a few studies in this line of inquiry, although their number is growing. Their most common approach is to examine the effect on reading time of including emojis at the beginning or at the end of the sentence or using them to replace a word within the sentence. The results show that people are sensitive to the congruency between the meaning of an emoji and the sentence, as is demonstrated by both behavioral (i.e., reading times) and electrophysiological (i.e., event-related potentials, or ERP) measures. Indeed, emojis that are congruent with the words they replace are read faster (Cohn et al., 2018) and elicit an N400 of lower amplitude (Tang et al., 2020; Weissman, 2019) than semantically incongruent emojis. Interestingly, the effects of emoji congruency on processing emerge in both early and late eye-tracking measures (Barach et al., 2021), as do the effects of word congruency (e.g., Dimigen et al., 2012). These findings suggest that emojis are semantically integrated within the text. In a related way, Robus et al. (2020) demonstrated that sentence reading times increase when emojis are in sentence-final positions, comparable to word-position effects in sentence processing (e.g., Kuperman et al., 2010).

The above findings show that the affective/semantic processing of emojis is analogous to other more extensively studied stimuli such as faces and words. Research using these stimuli has greatly benefited from studies in which the stimuli are characterized in terms of their affective meaning (e.g., Ferré et al., 2012; Hinojosa et al., 2016; Langner et al., 2010; Stadthagen-Gonzalez et al., 2018). In view of the emotional function of emojis, it is also interesting to characterize their affective meaning. Several steps in this direction have recently been taken in an attempt to match emojis with particular emotions. In some cases, the correspondence is established by researchers (i.e., human annotation, as in Novak et al., 2015). Other authors (e.g., Fernandez-Gavilanes et al., 2018) have relied on the official definition of emojis, which can be found on web pages such as Emojipedia. Still other authors have built emoji lexicons automatically, for instance by generating emoji vectors based on the frequency of their co-occurrence with emotional words (Kimura & Katsurai, 2017). These approaches have proved very useful and have been successfully applied in fields such as computer-based sentiment analysis, where affective reactions are detected and categorized based on the semantic analysis of written texts (e.g., Thelwall et al., 2012). A common limitation of these approaches, however, is that they do not rely on users’ perceptions (Rodrigues et al., 2018).

This is not a trivial issue, since a user’s interpretation does not always match the intended meaning of an emoji (i.e., the meaning intended by emoji developers, which can be found on the Unicode website) and there are likely to be differences between perceivers’ interpretations (Jaeger et al., 2019; Miller et al., 2016; Rodrigues et al., 2018). Moreover, a given emoji can be used to represent several meanings (Rodrigues et al., 2018). Also, emojis with the same meaning can have different visual representations across different operating systems and social platforms, while affective properties may be different across distinct visual representations (e.g., Franco & Fugate, 2020; Miller et al., 2016; Tigwell & Flatla, 2016).

In view of the above, it cannot be assumed that users interpret emojis and assess their affective properties according to their intended Unicode meaning. Normative studies in which users rate large sets of emojis in a series of relevant variables are therefore needed. To our knowledge, the only comprehensive study along these lines is that published by Rodrigues et al. in 2017. These authors developed the Lisbon Emoji and Emoticon Database (LEED), where a set of 238 stimuli (85 emoticons and 153 emoji) were rated by users in seven dimensions: aesthetic appeal, familiarity, visual complexity, semantic clarity, meaningfulness, valence, and arousal. Some of these variables were chosen from previous normative studies conducted with visual stimuli. Remarkably, two affective dimensions were also included: valence and arousal. According to dimensional models of emotion (Bradley & Lang, 1999), these are the two core dimensions in the description of the human affective space. The authors also included, when available, three variations of each emoji to examine any differences in ratings depending on the graphic representation used in the various platforms (Android, iOS, and Facebook). The results of the study showed that emojis were perceived as clearer, more familiar, and more meaningful than emoticons, probably because of the greater use of the former and the lesser use of the latter in recent years. Focusing on emojis, Rodrigues et al. found that most had a clear affective content, being either pleasant (positive) or unpleasant (negative). Moreover, most emojis were rated as being meaningful, clear, arousing, and highly familiar. A positive relationship between familiarity and valence was also observed, which indicates that pleasant emojis were perceived as more familiar than unpleasant ones. On the other hand, the comparison across platforms revealed differences in certain variables (aesthetic appeal, familiarity, clarity, and meaningfulness) but not in others (visual complexity, valence, and arousal). Finally, the authors also included an open-ended question that asked participants to provide the meaning of the emojis. In some cases, the meanings they provided were very similar to the intended Unicode meanings as well as very similar across the various formats. In other cases, the convergence was only partial. There were even numerous clear divergences, which illustrates that there is not always a direct correspondence in the meanings attributed to emojis across platforms or between the meanings attributed by users and the intended Unicode meanings.

A few studies have been conducted on a smaller scale since the publication of LEED. Jaeger et al. (2019), for example, asked participants to rate the affective properties (i.e., valence and arousal) of 33 face emoji and found the typical U-shaped relationship between valence and arousal (i.e., arousal increases as valence deviates from a neutral value, i.e., in positive and negative stimuli, in comparison to neutral stimuli). The similarity with the results obtained with pictures and words (e.g., Guasch et al., 2016; Libkuman et al., 2007) suggests that emojis can represent these two core dimensions of human affect like pictures and words do (Jaeger et al., 2019). In another study, Jones et al. (2020) examined gender differences in frequency of use, familiarity, and valence ratings in relation to 70 face emojis. In line with Rodrigues et al. (2018), these authors found a strong positive correlation between familiarity and valence. With regard to gender differences, women gave higher familiarity ratings overall and more negative valence ratings for negative emojis than men. Finally, Fischer and Herbert (2021) collected valence and arousal ratings for a small set of 18 face emojis, 18 emoticons, and 24 faces representing six human emotions. These authors found that emoticons were perceived as less emotional than emojis and faces but found no differences between emojis and faces. Also, like Jaeger et al., they found the typical U-shaped relationship between valence and arousal.

In summary, although previous normative studies have emphasized the need to address the affective features of emojis, they have collected ratings for only a small set of stimuli. To overcome this limitation, in the current study we present Emoji-SP. This database provides subjective norms for a large set of 1031 emojis in six dimensions: visual complexity, familiarity, frequency of use, clarity, emotional valence, and emotional arousal. It comprises a wide range of categories, including animals, body parts, clothes, drinks, emotions, facial expressions, family, food, professions, sports, and vehicles. This can be considered an improvement with respect to previous research because the normative studies published to date (Fischer & Herbert., 2021; Jaeger et al., 2019; Jones et al., 2020; Rodrigues et al., 2018) as well as most studies in the field (e.g., Comesaña et al., 2013; Erle et al., 2021; Kaye et al., 2021) have focused on face emojis. Collecting normative data on non-face emojis is highly relevant considering that their usage is widespread nowadays. In fact, when we checked the use of Twitter emojis in real time in Emojitracker (September 2021), we found that four of the top ten emojis were not faces. These data illustrate the widespread communicative use of non-face emojis.

The distinction between face and non-face emojis is also relevant from a theoretical point of view. Indeed, in a series of recent studies based on naturalistic Twitter examples and experimental evidence by Grosz et al. (2021) and Kaiser and Grosz (2021), it was argued that face emojis and non-face emojis differ in their semantic properties and should therefore be analyzed in a different manner. According to Kaiser and Grosz (2021), both face and non-face emojis involve anaphoric dependencies (i.e., they can be linked to the preceding linguistic context), although of different types. These authors argue for two types of emoji-text dependencies, related to referential dependencies known to exist in the linguistic domain: Face emojis resemble expressions (e.g., *wow*), which express affective states and attitudes of the speaker, while non-face emojis (mostly action emojis and objects) are interpreted based on principles of discourse coherence (e.g., they express relations like elaboration or explanation).

In view of the above, the characterization of a large set of face and non-face emojis in several relevant variables may have both theoretical and methodological implications. From a methodological point of view, it will facilitate the selection of well-characterized stimuli for studies in various fields. From a theoretical point of view, these studies will contribute to our knowledge of the affective and psycholinguistic functions served by the different types of emojis. Overall, the norms may help to further our knowledge of emoji functions, patterns of use and processing characteristics.

## Materials and methods

### Participants

A total of 1124 native Spanish speakers participated in this study. The sample size was selected considering those used in other normative studies, which have collected ratings for a similar number of stimuli and variables. All participants were students from Spanish universities: 54% from Universitat Rovira i Virgili (Tarragona, Spain), 42% from Universidad de Murcia (Murcia, Spain), and 4% from Universidad Complutense de Madrid (Madrid, Spain). Eighty-two participants were removed from the analyses because of atypical responses (see the data trimming procedure in the *Procedure* section). The average age of the remaining 1042 participants was 21.3 years (*SD* = 5.85, range = 17–59), of whom 910 were women (87.33% of the sample) and 132 were men (12.67% of the sample). Before rating the stimuli, the participants completed a brief questionnaire on the frequency with which they used social networks. Mean use was 1.71 (*SD* = 0.90) on a scale from 0 (never) to 3 (frequent, every day). All participants received academic credits for their participation and signed an informed consent document before the norming study began.

#### Materials

The database consists of 1031 emojis from Unicode Emoji version 13, published by the Unicode Foundation (http://unicode.org/emoji/charts/full-emoji-list.html). From this list, we selected a representative set of emojis while excluding those that were visually very similar (e.g., varying only in color), those that were idiosyncratic to a certain culture (e.g., Asian dishes that are unknown or little known in Western culture), those with an unclear distinction between the male and female versions, those related to signs and astrological symbols, etc., and those whose style of visual representation was different from most emojis in the list (e.g., emojis drawn with simple strokes and those with no color). Both male and female versions of the emojis were retained when the gender distinction was visually clear, especially when they referred to professions. The final emoji selection covers a wide range of categories including animals, body parts, clothes, drinks, emotions, facial expressions, family, food, professions, sports, and vehicles (see Appendix 14 for a full list of categories and the number of exemplars in each category).

#### Procedure

The 1031 emojis included in the final selection were presented in their Facebook version^1^. Participants rated the emojis on six dimensions: visual complexity, familiarity, frequency of use, clarity, emotional valence, and emotional arousal. The instructions (summarized) and the scale for each dimension are shown in Table 1. Full instructions can be found in Appendix 16.

**Table 1:** Instructions (summarized) and rating scale for each dimension collected in the study

Dimension	Instructions	Scale
Visual complexity	Visual complexity refers to the emoji’s visual features, not to the features of the concept to which it refers. The more visual features the emoji contains, the more visually complex it can be considered to be.	1 (very simple), 7 (very complex)^a^
Familiarity	Familiarity refers to how often the participant encounters or sees the emoji in his/her daily life. Emojis encountered more frequently are, therefore, more familiar.	1 (very unfamiliar), 7 (very familiar)^a^
Frequency of use	Frequency of use refers to how often the participant uses the emoji.	1 (I never use the emoji), 7 (I use the emoji very often)^a^
Clarity	Clarity refers to the relationship between the emoji and its meaning.	1 (The emoji does not represent the meaning at all), 7 (The emoji represents the meaning very well)^a,b^
Emotional valence	Emotional valence refers to the extent to which the emoji denotes something negative/unpleasant or something positive/pleasant.	1 (Very negative), 9 (Very positive)^a,c^
Emotional arousal	Emotional arousal refers to the extent to which the emoji denotes something passive/calm or something arousing/exciting.	1 (Very passive/calm), 9 (Very arousing/exciting) ^a,c^

^a^ Participants could choose the response “I don’t know this emoji” to indicate that they did not know the emoji (these responses were not included in the analyses). ^b^ For clarity ratings, a definition of each emoji was provided together with the scale. ^c^ Participants were provided with the Self-Assessment Manikin (SAM) (Bradley & Lang, 1999) to rate the emojis on the emotional dimensions

**Fig. 1:**
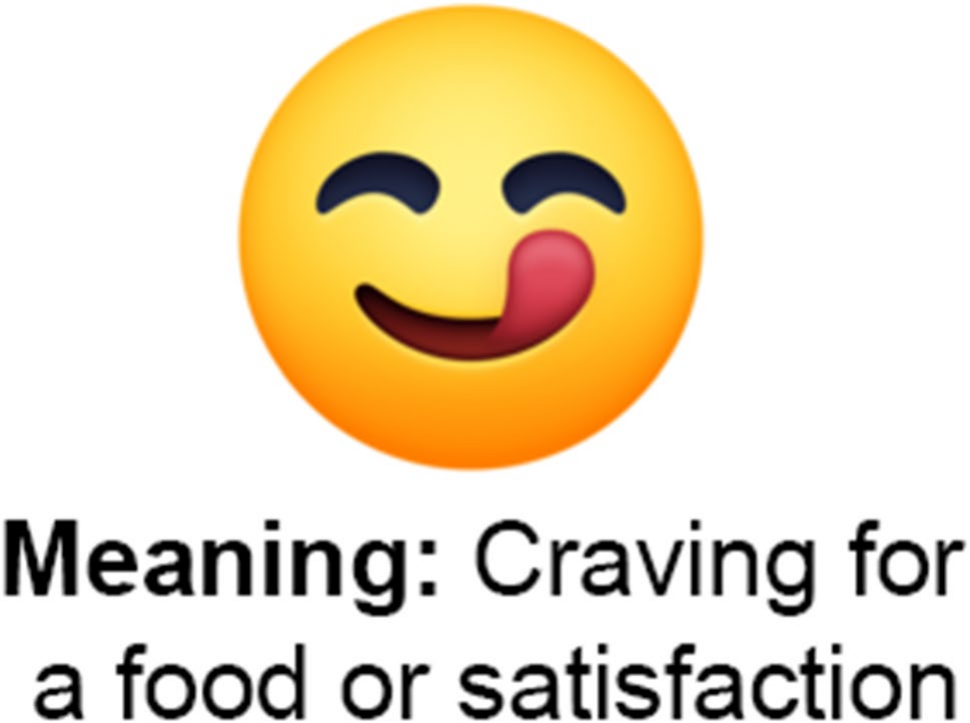
Example of an emoji with two meanings. Definitions were provided to participants in Spanish

We created five versions of the questionnaire for each dimension. Four versions included 205 emojis, while one version included 211. The questionnaires were created and administered online using TestMaker (Haro, 2012). The emojis were randomized in each questionnaire and displayed in png format with a size of 72 × 72 pixels. Each page of the questionnaire presented a list of 26 emojis (except the last page, where 23 or 29 were included, depending on the version of the questionnaire), the instructions for the dimension, and the rating scale (except the questionnaires for the clarity dimension, where a single emoji accompanied by its definition appeared on each page). The definitions were generated and translated into Spanish from the information provided by the website www.emojipedia.org and the description of each emoji provided by the Unicode Foundation. When emojis had more than one meaning, both meanings were included in the definition (see Fig. 1). In total, we identified 77 emojis with more than one meaning.

## Results

### Data trimming

Between 27 and 35 responses were obtained for each questionnaire (*M* = 31.22, *SD* = 1.84). Following the usual approach in this field (e.g., Pérez-Sánchez et al., 2021; Stadthagen-Gonzalez et al., 2018), responses whose ratings correlated poorly with the mean for all ratings on the same questionnaire were removed (i.e., *r <* 0.1; number of responses removed = 26). Correlations close to zero are assumed in this field to reflect idiosyncratic response patterns, while negative correlations indicate that the participant understood the rating scale in reverse order. We also removed eight responses with less than 50% of the ratings completed. Data trimming led to the exclusion of 34 responses. After removing these responses, we obtained an average of 28.94 valid responses per questionnaire (range = 24–30, *SD* = 1.71). Each emoji received an average of 28.30 valid ratings (range = 14–30, *SD* = 1.23). There was an average of 0.64 “don't know” responses for each emoji (range = 0–18, *SD* = 1.23). Only valid responses were included in the analyses and descriptive statistics described below.

### Reliability and validity

The interrater reliability of each questionnaire was measured by the intraclass correlation coefficient (ICC; Koo & Li, 2016) using the *psych* package (version Revelle, 2020) in R (version 4.0.2). We used two-way random effects based on the absolute agreement of multiple raters (2,*k*). All ICCs were statistically significant (all *p* < .001; *M* = .97, *SD* = .02, range = .92–.99).

To assess the validity of our ratings, we compared them with those from other normative studies (Jaeger et al., 2019; Jones et al., 2020; Novak et al., 2015; Rodrigues et al., 2018) by calculating the Pearson’s correlation on the dimensions shared between datasets. Seventy-six emojis were in common with Rodrigues et al. (2018). Of these, 63 were available in both their iOS and Android versions, while 13 were available only in their iOS version. The correlations with our ratings were: *r* = .34 (Android) and *r* = .61 (iOS) for visual complexity, *r* = .45 (Android) and *r* = .53 (IOS) for familiarity, *r* = .95 (Android) and *r* = .94 (iOS) for emotional valence, and *r* = .64 (Android) and *r* = .57 (iOS) for emotional arousal (all *p* < .007). We also identified 55 emojis that were in common with Jones et al. (2020), with a correlation of *r =* .55 for familiarity and *r =* .95 for emotional valence (both *p* < .001). The 26 emojis in common with Jaeger et al. (2019) showed a correlation of *r =* .96 (*p <* .001) for emotional valence. We also compared our emotional valence ratings with affective ratings obtained by other procedures, specifically the scores computed from the sentiment of tweets (Novak et al., 2015). There was a correlation of *r* = .60 (*p* < .001) between these sentiment scores and our emotional valence ratings for the 498 emojis in common. Finally, we also compared our frequency ratings with the real-time emoji usage frequency provided by the Emojitracker website (http://emojitracker.com/ [retrieved September 27, 2021]). A total of 661 emojis were found on Emojitracker, with a correlation of *r* = .61, *p <* .001.

### Description of the assessed variables

**Fig. 2:**
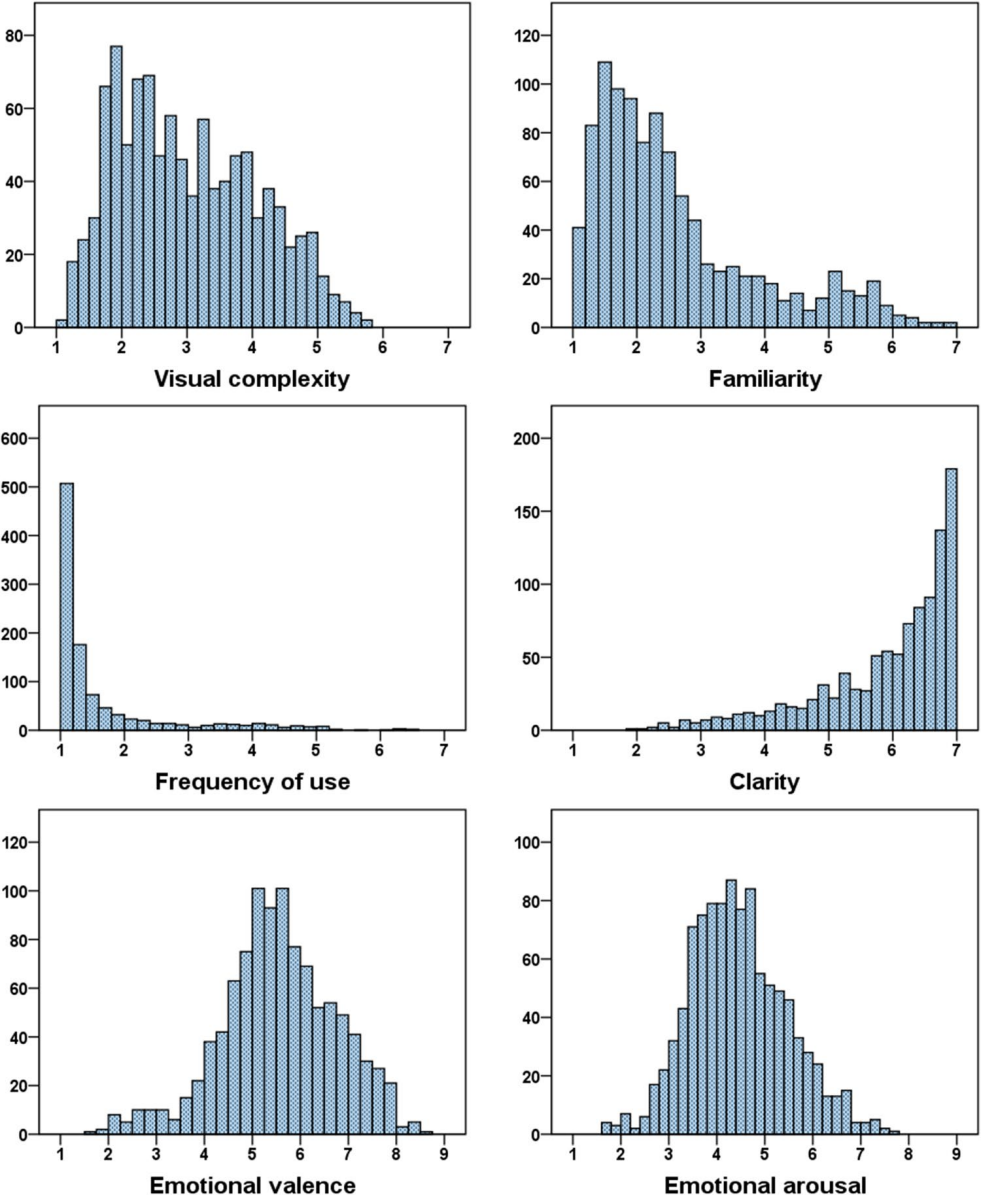
Distribution of the ratings of the assessed variables

The descriptive statistics and distribution of the dimensions collected in this study are shown in Table 2 and Fig. 2. Most emojis showed low familiarity and low frequency of use. Moreover, the data on visual complexity revealed that simple emojis (i.e., those with few visual attributes) predominate in the dataset. Regarding clarity, there appears to be a close relationship between emoji visual representation and meaning. With regard to the emotional dimensions, there are more positive and more neutral emojis than negative ones. Most stimuli in the dataset have a moderate level of arousal (i.e., they are neither excessively arousing nor excessively relaxing).

**Table 2:** Descriptive statistics of the assessed variables

Dimension	Mean	*SD*	Minimum	Maximum	Skewness	Kurtosis
Visual complexity	3.02	1.06	1.07	5.76	0.38	−0.82
Familiarity	2.59	1.28	1.00	6.87	1.21	0.67
Frequency of use	1.64	1.02	1.00	6.57	2.23	4.54
Clarity	5.91	1.08	1.95	7.00	−1.30	1.07
Emotional valence	5.55	1.21	1.63	8.63	−0.28	0.25
Emotional arousal	4.45	1.02	1.61	7.68	0.30	0.07

Apart from describing the entire dataset, we examined whether there were differences between distinct types of emojis in terms of the dimensions evaluated. To that end, we grouped the emojis into seven categories, following the categorization by Emojipedia (emojipedia.org): smileys and people (*n =* 381), animals and nature (*n* = 166), food and drink (*n* = 100), activity (*n* = 79), travel and places (*n* = 77), objects (*n =* 138), and symbols (*n =* 89). The means and *SDs* of each dimension for each category are shown in Table 3.

**Table 3:** Means and standard deviations of each dimension for each emoji category

Dimension	Category	*Mean*	*SD*
Frequency of use			
	Activity	1.15	0.17
	Animals and nature	1.46	0.52
	Food and drink	1.29	0.35
	Objects	1.20	0.39
	Smileys and people	2.15	1.37
	Symbols	1.68	1.09
	Travel and places	1.14	0.20
Familiarity			
	Activity	1.95	0.57
	Animals and nature	2.45	0.89
	Food and drink	2.36	0.84
	Objects	2.08	0.78
	Smileys and people	3.12	1.60
	Symbols	2.83	1.33
	Travel and places	1.89	0.60
Visual complexity			
	Activity	3.40	1.19
	Animals and nature	3.28	0.90
	Food and drink	2.76	0.75
	Objects	2.52	0.83
	Smileys and people	3.22	1.11
	Symbols	1.91	0.48
	Travel and places	3.61	0.88
Clarity			
	Activity	6.32	0.71
	Animals and nature	6.01	1.36
	Food and drink	6.58	0.58
	Objects	5.42	1.12
	Smileys and people	5.84	0.97
	Symbols	5.61	1.13
	Travel and places	6.01	1.00
Emotional arousal			
	Activity	4.72	0.93
	Animals and nature	4.18	1.12
	Food and drink	4.01	0.64
	Objects	4.35	0.93
	Smileys and people	4.62	1.06
	Symbols	4.58	0.91
	Travel and places	4.55	1.12
Emotional valence			
	Activity	5.75	0.69
	Animals and nature	5.86	0.91
	Food and drink	5.99	0.83
	Objects	5.07	0.95
	Smileys and people	5.46	1.49
	Symbols	5.48	1.29
	Travel and places	5.44	0.98

We performed a series of unifactorial analyses of variance (ANOVAs) to compare the mean ratings of each dimension between categories. Significant differences were observed in all dimensions: frequency of use, *F*(6, 1023) = 34.38, *MSE =* 30.15, *p <* .001*,* familiarity, *F*(6, 1023) = 26.06, *MSE =* 37.37, *p <* .001*,* visual complexity, *F*(6, 1023) = 38.73, *MSE =* 35.72, *p <* .001*,* clarity, *F*(6, 1023) = 16.29, *MSE =* 17.42, *p <* .001*,* emotional arousal, *F*(6, 1023) = 8.50, *MSE =* 8.54, *p <* .001*,* and emotional valence, *F*(6, 1023) = 8.96, *MSE =* 12.52, *p <* .001. The results showed that emojis in the smileys and people category were used more frequently and were more familiar than emojis in the other categories. Emojis of the symbols category, on the other hand, had higher frequency and familiarity ratings than those of the activity, objects, and travel and places categories. The emojis of the travel and places category, in turn, had the highest ratings on visual complexity (although the differences with the animals and activity categories were not significant); conversely, the emojis of the symbols category showed lower visual complexity ratings than the rest of categories. As for the clarity dimension, the emojis in the food and drink categories obtained the highest ratings (but without significant differences with the activity category). In contrast, the emojis in the objects category had lower clarity ratings than the other categories (except for those belonging to the symbols category). Regarding the emotional dimensions, emojis in the food and drink category were the least arousing (although the differences from the animals and nature and the objects categories were not significant) and those with the highest valence ratings (except for the comparison of activity versus animals and nature and symbols categories). Moreover, the emojis of the objects category showed the lowest valence ratings (but without significant differences from the symbols and travel and places categories).

In addition, we compared the emojis representing faces (smileys, *n* = 112) with the rest of the emojis. The results of the *t*-tests showed significant differences between both types of emojis in all dimensions, indicating that faces were more familiar (*M* = 4.89 vs *M* = 2.31), *t*(1029) = 25.76, *p* < .001, more frequently used (*M* = 3.66 vs *M* = 1.39), *t*(1029) = 30.60, *p* < .001, less visually complex (*M* = 2.55 vs *M* = 3.08), *t*(1029) = 5.04, *p* < .001, less clear (*M* = 5.42 vs *M* = 5.97), *t*(1029) = 5.19, *p* < .001, more emotionally arousing (*M* = 5.34 vs *M* = 4.34), *t*(1029) = 10.16, *p* < .001, and of a more negative valence (*M* = 4.96 vs *M* = 5.62), *t*(1029) = 5.49, *p* < .001 than the other emojis.

### Age and gender effects

We explored whether age and gender had any influence on ratings (the mean ratings for men and women in the six dimensions are presented in Table 4). A series of regression analyses were performed, where the age and gender of the participants were entered as predictors of the ratings of each dimension, in addition to the questionnaire version to which they responded (the reason to include this variable was that the difference in the number of male and female participants was larger in some versions of the questionnaire than in others). There was a gender effect in clarity ratings, *R*^2^ = .03, *F*(1, 148) = 4.27, *p* = .041, where women gave higher ratings than men, *b* = 0.21, *t* = 2.07, *p* = .041. There was also a gender effect in visual complexity ratings, *R*^2^ = .03, *F*(1, 135) = 2.68, *p* = .039, indicating that women rated emojis as visually more complex than men, *b* = 0.42, *t =* 2.09, *p* = .039. Regarding age, the only significant effect was on emotional arousal ratings, *R*^2^ = .03, *F*(1, 143) = 3.99, *p* = .048, indicating that older participants evaluated emojis as less activating than younger ones, *b* = −0.04, *t =* 2.00, *p* = .048.

**Table 4:** Means and standard deviations for each dimension in men and women

Dimension	Women (Mean)	Women (*SD*)	Men (Mean)	Men (*SD*)
Visual complexity	3.05	0.79	2.63	0.73
Familiarity	2.66	1.04	2.36	1.05
Frequency of use	1.67	0.39	1.54	0.38
Clarity	5.94	0.42	5.73	0.48
Emotional valence	5.58	0.56	5.38	0.62
Emotional arousal	4.51	1.21	4.15	1.38

### Relationships between the assessed variables

Pearson correlations between the variables assessed in this study are shown in Table 5.

**Table 5:** Bivariate correlations between variables

	Visual complexity	Frequency of use	Familiarity	Clarity	Emotional valence
Frequency of use	−.28^***^				
Familiarity	−.36^***^	.89^***^			
Clarity	.22^***^	−.18^***^	−.20^***^		
Emotional valence	.09^**^	.08^*^	.13^***^	.05	
Emotional arousal	.01	.26^***^	.27^***^	.01	−.35^***^

^*^ *p* < .05, ^**^ *p* < .01, ^***^ *p* < .001

Familiarity and frequency of use are highly correlated. Both these variables showed a significant correlation with all the other variables. Also, the size and sign of these correlations were almost identical for familiarity and frequency of use, i.e., a negative small-to-moderate correlation with visual complexity, a negative small correlation with clarity, a small positive correlation with emotional valence, and a small-to-moderate positive correlation with emotional arousal.

**Fig. 3:**
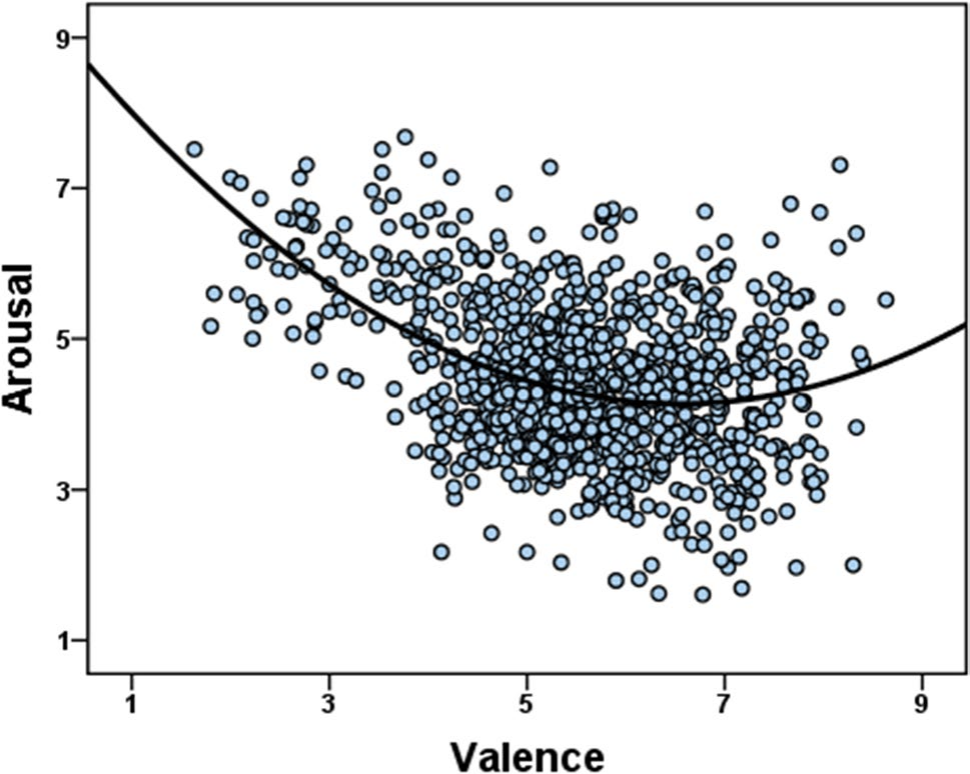
Relationship between the ratings of emotional arousal and emotional valence

With regard to the two affective variables, there was a moderate negative correlation between emotional valence and emotional arousal. Moreover, since previous studies with emojis (Jaeger et al., 2019; Fischer & Herbert, 2021) reported a U-shaped relationship between valence and arousal, we examined this issue using a quadratic regression. We found that the relationship between emotional valence and arousal fits a quadratic trend, *R*^2^ = .19, *F*(2, 1028) = 120.49, *p* < .001, better than a linear one, *R*^2^ = .12, *F*(1, 1029) = 141.34, *p* < .001 (these regression analyses were performed using IBM SPSS, version 23.0, IBM Corp., USA); i.e., the more positive/negative an emoji is, the more arousing it is perceived (hence, the so-called U-shaped or boomerang relationship between valence and arousal, see Fig. 3).

## Discussion

The aim of this study was to provide subjective norms for a large set of emojis from a wide range of categories. The norms contain ratings for 1031 emojis in six evaluative dimensions: visual complexity, familiarity, frequency of use, clarity, emotional valence, and emotional arousal.

The ratings show high indices of interrater reliability. Validity is also high, as is indicated by comparison with the few normative studies published so far. Regarding the emotional dimensions, the correlations between the valence ratings across studies were very strong. Interestingly, the correlation with affective (sentiment) scores obtained using a different procedure (Novak et al., 2015), though more moderate, was also strong. This indicates a high level of agreement between users in assigning an affective polarity to emojis. Agreement was lower in relation to arousal. Although the correlations across datasets were still strong for this variable, their magnitude was smaller than for valence. This is in line with results from the few studies that have collected affective ratings for emojis (e.g., Jaeger et al., 2019) as well as with results obtained using other types of affective stimuli (e.g., Pérez-Sánchez et al., 2021). According to Pérez-Sánchez et al. (2021), one reason for this common finding may be the disparity between raters in their understanding of the concept of arousal, since some may understand arousal as being closely related to valence (i.e., the intensity of the pleasant or unpleasant experience), while others may understand it as a completely separate dimension from valence (i.e., a feeling of activation, excitation, etc.).

With respect to the other variables, the correlations across databases for visual complexity and familiarity were moderate. This may be due to the small number of shared stimuli between studies or to the fact that the versions of emoji used by Rodrigues et al. (2018; iOS and Android) and Jones et al. (2020; iOS) were different from those used here (i.e., Facebook). Another reason for this moderate correlation with familiarity may be related to cultural differences. Evidence in this direction was obtained by Jones et al., who compared their ratings to those of Rodrigues et al. Interestingly, although the correlation was moderate (*p* = .51), several emojis showed important differences between the Portuguese (Rodrigues et al., 2018) and the U.S. (Jones et al., 2020) samples. Jones et al. pointed out that cultural differences may be valence-dependent, as they found a higher agreement for positive emojis than for negative ones. This suggests that positive emojis tend to be more familiar across cultures, while negative ones are more variable when it comes to familiarity. To address this issue, we selected two subsets of emojis according to their emotional valence ratings, i.e., negative (valence < 4) and positive (valence > 6). We followed similar categorization criteria to those used in previous studies with words (e.g., Ferré et al., 2012; Huete-Pérez et al., 2019). Like Jones et al., we obtained higher correlations with the familiarity ratings of Rodrigues et al.^2^ and Jones et al. for positive emojis (*r* = 0.65 and *r* = 0.68, respectively) than for negative ones (*r* = 0.49 and *r* = 0.50, respectively). Finally, there was a strong correlation between our frequency ratings (a subjective measure from a Spanish sample) and the data on real-time emoji usage obtained from the Emojitracker website (an objective measure from the global users of Twitter). This suggests that participants’ subjective ratings are a good indicator of the current worldwide emoji use.

In addition to examining the reliability and validity of our ratings, we characterized the emojis in the database in relation to the assessed variables. Most emojis showed a low familiarity and frequency of use, which suggests that users tend to encounter or use only a small set of the total number of emojis analyzed. To some extent this result contrasts with that observed by Rodrigues et al. (2018), who reported a higher familiarity rating (*M* = 4.43) than we obtained in this study (*M* = 2.59). Note that Rodrigues et al. selected only 153 emojis for evaluation, so these were probably the most representative of all those used in social networks. In support of this assumption, we confirmed that the average familiarity rating of the 76 emojis shared with that study was higher (M = 5.25) than that of the overall dataset (see above) and very similar to that of Rodrigues et al. for the same subset of items (*M* = 5.00). Consistent with that, the analyses carried out with our dataset revealed differences between the distinct categories of emojis. Specifically, face emojis were rated as more familiar and more frequently used than emojis in all the other categories (i.e., non-face emojis). Although there were also small differences between some types of non-face emojis (e.g., emojis in the symbol category showed higher ratings than the other categories), the use/knowledge of face emojis clearly stands out from the others. These findings suggest that face emojis are more typically used in communicative interactions than non-face emojis. Considering the expressive function of face emojis (Kaiser & Grosz, 2021), this seems to indicate that emojis are used in social networks mostly to express affective states and attitudes.

On the other hand, like those of Rodrigues et al. (2018), our data on visual complexity show that, overall, emojis with simple visual representations (i.e., those with few visual attributes) are the most common. However, there were differences between emoji categories in this dimension: Face emojis were rated as less visually complex than non-face emojis. Among the latter, the emojis perceived as less visually complex were those belonging to the symbols category while the more complex were those in the travel and places category. Regarding clarity, users’ ratings reveal that the visual representations of the emojis in the dataset are closely related to their meaning, although there were again differences between categories. Among non-face emojis, those in the food and drink category were considered as more closely related to their meaning, while those in the objects category were less clear. Interestingly, the comparison between face and non-face emojis revealed that the former were slightly less clear than the latter. Considering the expressive function of face emojis (Kaiser & Grosz, 2021), this may indicate that in some cases their correspondence with a particular affective state and attitude is not unequivocal.

Two issues deserve mention in relation to the emotional dimensions. Firstly, most emojis have a moderate arousal level (i.e., they are neither excessively arousing nor excessively relaxing), in agreement with Rodrigues et al. (2018). In fact, the average rating for emotional arousal (*M* = 4.45) was similar to that reported by these authors (*M* = 4.84). As in the previously discussed dimensions, there were differences in arousal ratings between the different types of emojis. Concretely, there were slight differences within non-face emojis, with those in the food and drink category being perceived as less arousing than the others, while face emojis were clearly considered as more arousing than non-face emojis. Secondly, also in agreement with Rodrigues et al. (2018), many emojis convey a positive or neutral emotional valence rather than a negative one. Moreover, few emojis have an extreme valence, whether negative or positive. It should be noted, however, that the average valence rating (*M* = 5.55) was higher than that in Rodrigues et al. (2018) (*M* = 4.08; difference = 1.47). As mentioned earlier with regard to familiarity, these discrepancies between studies may be due to the different versions of emojis used (i.e., Android and iOS versions in the study by Rodrigues et al. and Facebook in the present study) or to the large difference in the number of emojis evaluated (238 in Rodrigues et al. and 1031 in the present study, i.e., over four times more). Another reason may be the set of emojis included in the study by Rodrigues et al. (mostly face emojis), a possibility supported by the fact that the difference between studies in average emotional valence decreases if we consider only the ratings of the 76 emojis in common (*M* = 4.77 in this study vs *M =* 3.95 in Rodrigues et al.; difference = 0.82). In line with that, we found that face emojis were perceived as more negative than non-face emojis. This makes sense considering that face emojis convey a wide range of human emotions, from positive to negative (e.g., Cherbonnier & Michinov, 2021). Such variability is not expected in non-face emojis. In fact, the difference found here between face and non-face emojis in valence and arousal ratings is in line with the distinct types of functions served by these two categories of emojis, with face emojis being more strongly related to affective expression (Kaiser & Grosz, 2021).

The exploration of age and gender effects on our data revealed only modest influences. Women rated emojis as clearer and more visually complex than men. These results partly agree with those of Rodrigues et al. (2018), who also found higher clarity ratings for women, but who did not find any difference between males and females in terms of visual complexity. We did not find a gender effect either in frequency of use, familiarity or valence ratings, as other authors have reported (e.g., Jones et al., 2020; Prada et al., 2018). Regarding age, the only variable showing an effect was emotional arousal, where older participants rated emojis as less activating than younger participants. There was not any effect of age on frequency of use, in contrast to previous results (e.g., Prada et al., 2018; Settanni & Marengo, 2015). It should be noted that the analyses involving age and gender are exploratory in nature, considering that it was not among the main aims of the study to examine such variables. Therefore, the results should be interpreted with caution, given that the age distribution of the sample was very narrow (90% of the participants were between 17 and 25 years old) and that there was a large disproportion between the number of female participants and male participants (87% and 13%, respectively). Only a study involving a wide range of age groups and the same proportion of genders may shed light on these issues.

Finally, we also examined the pattern of relationships between the variables in the dataset. Our results showed that familiarity and frequency of use were highly correlated; i.e., the most familiar emojis are also those most often used. Both variables showed a significant correlation with all the other variables. The correlation was positive with emotional valence and arousal and negative with visual complexity and clarity. This suggests that the most familiar and frequently used emojis tend to be visually simpler, more emotionally arousing, and more positively valenced. The latter correlation indicates that people use and encounter more positive emojis than negative ones in their interactions, which is consistent with the fact that people use emojis more when they are in a good mood (Konrad et al., 2020). The positive correlation between emotional valence and familiarity agrees with those reported by Jones et al. (2020) (*r =* .45) and Rodrigues et al. (2018) (*r =* .25). It also agrees with studies conducted with other types of affective stimuli, such as words (e.g., Citron et al., 2012; Verheyen et al., 2020). This means that, like positive words are used more often than negative words (the so-called positivity bias, described in multiple languages, Dodds et al., 2015), positive emojis are more frequently used than negative emojis. Also in line with Rodrigues et al. are the correlations obtained between familiarity and the other variables (i.e., −.19 with visual complexity and .31 with emotional arousal). The only exception was in relation to clarity. Note that this variable was assessed differently in each study: we asked our participants to rate the extent to which each emoji represented its intended meaning (which we provided), while Rodrigues et al. asked them to rate how clearly each emoji conveys an emotion/meaning (which was not made explicit). This methodological difference suggests that caution is needed when comparing the results on clarity between the two studies. Methodological differences may also explain the diverging pattern of correlations between clarity and visual complexity, which were positive here (*r =* .22) and negative in Rodrigues et al. (*r =* −.18). Another variable which might influence the pattern of correlations (and the discrepancy with previous studies regarding certain variables) is the ambiguous meaning of some emojis (i.e., emojis that have more than one meaning). We consider this unlikely given the small number of ambiguous emojis in our dataset (77 out of 1031, i.e., 7.45%). To rule out this possibility, we repeated the correlation analyses between variables after excluding the ambiguous emojis, finding the same pattern of results as with the entire dataset^3^. Nevertheless, it would be interesting to examine ambiguous emojis in more detail in future research. Concretely, it may have been difficult for participants to give a single rating (e.g., valence ratings) for emojis that have more than one meaning. A different approach, where participants are asked to rate each meaning of the emoji separately, may be useful. Such an approach has recently been used with ambiguous words (Huete-Pérez et al., 2020), showing that the best predictor of global valence ratings (i.e., ratings provided when an ambiguous word, like *cataract*, is presented in isolation, without disambiguating its meaning) is a combination of the ratings of each separate meaning (i.e., waterfall and eye disease) that takes into consideration their relative dominance. It would be interesting to examine whether the same is true for ambiguous emojis.

A final point deserving to be mentioned here is the pattern of correlations observed between the two affective variables. In line with previous studies with emojis (Fischer & Herbert, 2021; Jaeger et al., 2019), we found a U-shaped relationship between valence and arousal. This suggests that emojis’ arousal generally increases in line with their affective charge; i.e., emojis that are more negative or more positive are also perceived to be more arousing. The same pattern has been observed repeatedly in numerous studies conducted with stimuli such as images (e.g., Grun & Scheibe, 2008), faces (e.g., Schmidtmann et al., 2020), words (e.g., Guasch et al., 2016), or figurative expressions (e.g., Gavilán et al., 2021). It appears, therefore, that emojis represent the dimensions of valence and arousal in a similar way to other types of stimuli. Like these other stimuli, therefore, they can be used to study human affective processing. Recent findings support this possibility. For instance, face emojis have shown to produce affective responses (measured with affective ratings, e.g., Fischer & Herbert, 2021; Gantiva et al., 2021), neural responses (measured with event-related potentials, e.g., Gantiva et al., 2020) and psychophysiological responses (measured with the startle reflex, Aluja et al., 2020, and with electromyographic recordings, Gantiva et al., 2021) that are like those produced by human faces expressing emotions. Furthermore, emojis, like words, have been found to modulate the affective processing of words with which they co-occur, both at a behavioral and at a neural level (Comesaña et al., 2013; Pfeifer et al., 2022; Yang et al., 2021). We may expect in the coming years an expansion in the number of studies devoted to the comparison of the affective processing of emojis with other types of affective stimuli. A large normative emoji dataset will undoubtedly facilitate such research.

## Conclusions

In this paper we have presented Emoji-SP, which contains ratings for 1031 emojis in six evaluative dimensions. This is the largest emoji normative study so far conducted that relies on subjective ratings. The scientific literature reveals differences between emojis’ intended meanings and users’ interpretations. This resource thus complements other approaches that have relied on official definitions or researchers’ intuitions to characterize emoji. Emoji-SP will prove valuable for researchers since it provides them with a data-driven way to select emojis that are well characterized in affective and non-affective dimensions. It may have a significant impact given the widespread use of emojis and the scientific interest they have attracted in fields such as behavioral science, computer science, communication, linguistics, medicine, and psychology, to name but a few.

## Figures and Tables

**Fig. 4:**
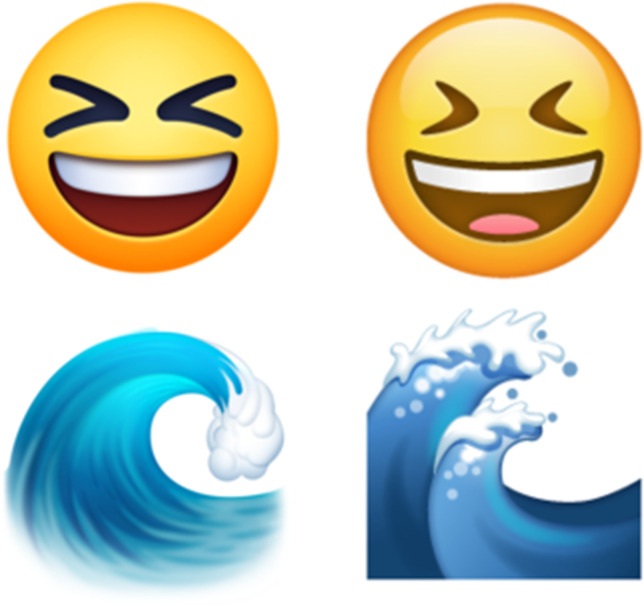
Examples of different versions of two emojis: Facebook emojis (on the left) and WhatsApp emojis (on the right)

## Appendix 1: List of emoji categories and number of items in each category

**Table:**

Category	Number of emojis	Percentage
animal-amphibian	1	0.10
animal-bird	15	1.45
animal-bug	10	0.97
animal-mammal	58	5.63
animal-marine	9	0.87
animal-reptile	8	0.78
arts & crafts	4	0.39
audio/visual symbol	3	0.29
award-medal	5	0.48
body-parts	15	1.45
book-paper	13	1.26
cat-face	9	0.87
clothing	41	3.98
computer	8	0.78
dishware	5	0.48
drink	15	1.45
emotion	34	3.30
event	13	1.26
face-affection	8	0.78
face-concerned	23	2.23
face-costume	8	0.78
face-glasses	3	0.29
face-hand	4	0.39
face-hat	2	0.19
face-negative	7	0.68
face-neutral-skeptical	10	0.97
face-sleepy	5	0.48
face-smiling	13	1.26
face-tongue	6	0.58
face-unwell	11	1.07
family	36	3.49
food-Asian	10	0.97
food-fruit	17	1.65
food-marine	5	0.48
food-prepared	30	2.91
food-sweet	13	1.26
food-vegetable	13	1.26
game	12	1.16
hairstyle	4	0.39
hand-fingers-closed	6	0.58
hand-fingers-open	5	0.48
hand-fingers-partial	7	0.68
hand-prop	3	0.29
hand-single-finger	6	0.58
hands	6	0.58
hotel	1	0.10
household	15	1.45
light & video	11	1.07
lock	4	0.39
mail	11	1.07
medical	5	0.48
money	6	0.58
monkey-face	3	0.29
music	6	0.58
musical instrument	7	0.68
office	15	1.45
other-object	2	0.19
other-symbol	2	0.19
person	21	2.04
person-activity	26	2.52
person-fantasy	21	2.04
person-gesture	18	1.75
person-resting	4	0.39
person-role	50	4.85
person-sport	26	2.52
person-symbol	3	0.29
phone	4	0.39
place-building	16	1.55
place-geographic	2	0.19
place-map	5	0.48
place-other	14	1.36
place-religious	4	0.39
plant-flower	9	0.87
plant-other	11	1.07
religion	1	0.10
science	6	0.58
sky & weather	28	2.72
sound	9	0.87
sport	22	2.13
time	27	2.62
tool	9	0.87
transport-air	8	0.78
transport-ground	27	2.62
transport-sign	12	1.16
transport-water	5	0.48
warning	2	0.19
writing	1	0.10
zodiac	13	1.26

## Appendix 2: Pilot study

We conducted a pilot study to decide which version of emojis we should use in the normative study (Facebook or WhatsApp). We selected a subset of 205 emojis from each version (see Appendix Fig. 4). Two questionnaires were created for each version, one to assess frequency of use and the other to assess familiarity. We used the same instructions and procedure as in the normative study to evaluate these dimensions (see *Materials and methods*). Thirty responses were obtained for each questionnaire. The correlations between the ratings of the two versions (*n* = 205) were very high (*r* = .95 for frequency, and *r* = .92 for familiarity). However, paired *t*-tests showed differences between ratings (both *p* < .01; see Table 5). According to the participants’ ratings, Facebook emojis are more familiar and used more often than WhatsApp emojis. In view of these results, we chose Facebook emojis for our normative study.

Appendix Table 6

**Table 6:** Ratings of Facebook and WhatsApp emojis (*n* = 205)

Dimension	Facebook		WhatsApp		Differences	
	Mean	*SD*	Mean	*SD*	*t*	*p*
Frequency of use	1.59	0.99	1.53	0.98	2.77	.006
Familiarity	2.51	1.22	2.08	1.27	12.56	<.001

## Appendix 3: Instructions for each dimension (Spanish)

Below are the instructions in Spanish (as seen by the participants), followed by their English translations.

### Visual complexity

A continuación, se te presentará una lista de *emojis* (dibujos o signos que expresan una emoción o idea). Te pedimos que evalúes su complejidad visual. La complejidad visual se refiere a las características visuales del *emoji*, no a las características del concepto al que se refiere. Cuantos más detalles visuales contenga el *emoji*, más complejo será. Para realizar tu valoración, usarás una escala continua que va de 1 a 7. Si consideras que el *emoji* es muy simple, marca un 1. En cambio, si consideras que es muy complejo, marca un 7. Puedes usar cualquiera de los valores intermedios de la escala. Acuérdate de responder a todos los *emojis*. Si hay alguno que no conozcas, marca la opción “no conozco el *emoji*”.

### Familiarity

A continuación, se te presentará una lista de *emojis* (dibujos o signos que expresan una emoción o idea). Te pedimos que evalúes su familiaridad. La familiaridad se refiere a la frecuencia con la que te encuentras o ves el *emoji* en tu vida cotidiana. Aquellos *emojis* que te encuentres más frecuentemente serán más familiares. Importante: no nos referimos a la frecuencia con la que usas el *emoji*, sino a la frecuencia con la que te lo encuentras en las distintas plataformas digitales. Para realizar tu valoración, usarás una escala continua que va de 1 a 7. Si para ti el *emoji* no es nada familiar, marca un 1. En cambio, si para ti es muy familiar, marca un 7. Puedes usar cualquiera de los valores intermedios de la escala. Acuérdate de responder a todos los *emojis*. Si hay alguno que no conozcas, marca la opción “no conozco el *emoji*”.

### Frequency of use

A continuación, se te presentará una lista de *emojis* (dibujos o signos que expresan una emoción o idea). Te pedimos que evalúes con qué frecuencia usas cada *emoji*. Importante: no nos referimos a la frecuencia con la que te encuentras o ves este *emoji* (o a la frecuencia con la que lo usan los demás), sino a cuánto lo usas tú. Para realizar tu valoración, usarás una escala continua que va de 1 a 7. Si no usas nunca el *emoji*, marca un 1. En cambio, si usas muy frecuentemente el *emoji*, marca un 7. Puedes usar cualquiera de los valores intermedios de la escala. Acuérdate de responder a todos los *emojis*. Si hay alguno que no conozcas, marca la opción “no conozco el *emoji*”.

### Clarity

A continuación, se te presentará una lista de *emojis* (dibujos o signos que expresan una emoción o idea) junto con su significado. Te pedimos que evalúes la relación entre el *emoji* y su significado en una escala de 1 a 7. Si consideras que el *emoji* no representa en absoluto el significado al que se refiere, marca un 1. En cambio, si consideras que el *emoji* representa muy bien el significado al que se refiere, marca un 7. Puedes usar cualquiera de los valores intermedios de la escala. Acuérdate de responder a todos los *emojis*. Si hay alguno que no conozcas, marca la opción “no conozco el *emoji*”.

### Emotional valence

A continuación, se te presentará una lista de *emojis* (dibujos o signos que expresan una emoción o idea). Te pedimos que los evalúes usando el Maniquí de Auto-Evaluación (MAE) que aparece en la imagen. Observa que el MAE dispone de una escala continua que va desde 1 hasta 9. Utiliza estos valores para evaluar cada *emoji* en la dimensión AGRADO de acuerdo con los siguientes criterios:

Si el *emoji* te hace sentir completamente triste, lo evaluarás con un 1. Si te hace sentir completamente alegre, lo evaluarás con un 9. Si no te hace sentir ni alegre ni triste, sino neutral, lo evaluarás con un 5. Puedes utilizar otros valores si el *emoji* te hace sentir un poco triste (3) o un poco alegre (7). Observa que también puedes evaluar tu nivel de alegría o tristeza utilizando otros valores (2, 4, 6, 8) situados entre las figuras. Acuérdate de responder a todos los *emojis*. Si hay alguno que no conozcas, marca la opción “no conozco el *emoji*”.

### Emotional arousal

A continuación, se te presentará una lista de *emojis* (dibujos o signos que expresan una emoción o idea). Te pedimos que los evalúes usando el Maniquí de Auto-Evaluación (MAE) que aparece en la imagen. Observa que el MAE dispone de una escala continua que va desde 1 hasta 9. Utiliza estos valores para evaluar cada *emoji* en la dimensión ACTIVACIÓN de acuerdo con los siguientes criterios:

Si el *emoji* te hace sentir completamente calmado (es decir, muy desactivado o muy relajado) lo indicarás mediante un 1. Cuando el *emoji* te haga sentir completamente activado (es decir, muy activado o muy despierto), lo indicarás con un 9. Utiliza un 5 si el *emoji* no te calma ni te activa, es decir, si lo evalúas como neutral en esta dimensión. Puedes utilizar otros valores si el *emoji* te hace sentir un poco relajado (3) o un poco activado (7). Observa que también puedes evaluar tu nivel de calma o activación utilizando otros valores (2, 4, 6, 8) situados entre las figuras. Acuérdate de responder a todos los *emojis*. Si hay alguno que no conozcas, marca la opción “no conozco el *emoji*”.

## Instructions for the dimensions (English)

### Visual complexity

Below you will find a list of emojis (i.e., pictures or signs that express an emotion or idea). Please assess their visual complexity. Visual complexity refers to the visual features of the emoji, not the features of the concept it refers to. The more visual details the emoji contains, the more complex it is. Use a scale from 1 to 7 for your rating. If you think the emoji is very simple, select 1. If you think it is very complex, select 7. Remember to rate every emoji. If there are any you do not know, select the option “I don’t know this emoji”.

### Familiarity

Below you will find a list of emojis (i.e., pictures or signs that express an emotion or idea). Please assess their familiarity. Familiarity refers to how often you encounter or see the emoji in your daily life. Emojis you encounter more frequently will be more familiar to you. This does not refer to how often you use the emoji but how often you encounter it in the various social networks. Use a scale from 1 to 7 for your rating. If the emoji is not at all familiar to you, select 1. If it is very familiar to you, select 7. Remember to rate every emoji. If there are any you do not know, select the option “I don’t know this emoji”.

### Frequency of use

Below you will find a list of emojis (i.e., pictures or signs that express an emotion or idea). Please evaluate how often you use each emoji. This does not mean how often you encounter or see this emoji (or how often others use it), but how often you use it. Use a scale from 1 to 7 for your rating. If you never use the emoji, select 1. If you use the emoji very often, select 7. Remember to rate every emoji. If there are any you do not know, select the option “I don’t know this emoji”.

### Clarity

Below you will find a list of emojis (i.e., pictures or signs that express an emotion or idea). Please rate the relationship between the emoji and its meaning on a scale of 1 to 7. If you feel the emoji does not at all represent the meaning it refers to, select 1. If you feel the emoji represents the meaning it refers to very well, select 7. Remember to rate every emoji. If there are any you do not know, select the option “I don’t know this emoji”.

### Emotional valence

Below you will find a list of emojis (i.e., pictures or signs that express an emotion or idea). Please evaluate these emojis using the Self-Assessment Manikin (SAM) shown in the image. Notice that the SAM has a scale from 1 to 9. Use this scale to evaluate each emoji in the VALENCE dimension according to the following criteria:

If the emoji makes you feel completely sad, select 1. If it makes you feel completely happy, select 9. If it makes you feel neutral rather than happy or sad, select 5. You can select other ratings if the emoji makes you feel quite sad (3) or quite happy (7). Note that you can also rate your level of happiness or sadness by selecting the other numbers (2, 4, 6, 8) located between the images. Remember to rate every emoji. If there are any that you do not know, select the option “I don’t know this emoji”.

### Emotional arousal

Below you will find a list of emojis (i.e., pictures or signs that express an emotion or idea). Please evaluate these emojis using the Self-Assessment Manikin (SAM) shown in the image. Notice that the SAM has a scale from 1 to 9. Use this scale to evaluate each emoji in the AROUSAL dimension according to the following criteria:

If the emoji makes you feel completely calm (i.e., very relaxed), select 1. If it makes you feel completely aroused (i.e., wide awake), select 9. Select 5 if the emoji neither calms you down nor arouses you, i.e., if you feel neutral in this dimension. Use other numbers if the emoji makes you feel quite relaxed (3) or quite aroused (7). Note that you can also rate your level of calmness or arousal by selecting the other numbers (2, 4, 6, 8) located between the images. Remember to rate every emoji. If there are any you do not know, select the option “I don’t know this emoji”.

## Footnotes

1 A pilot study was conducted in which 63 participants rated the frequency and familiarity of a subset of emojis from two of the most frequently used social networks: Facebook and WhatsApp (see Appendix B for a detailed description). The results of this pilot study showed that Facebook emojis were more familiar and more often used. We therefore chose to conduct the normative study using Facebook emojis.

2 We only report correlations for the iOS version, as there were not enough stimuli in the Android version to calculate reliable correlations.

3 We would like to thank an anonymous reviewer for this suggestion.
